# Contagious Diseases

**Published:** 1872-10

**Authors:** 


### CONTAGIOUS DISEASES

Of the ordinary kind are such as are communicated by contact, by coming near enough a sick person to breathe into the lungs, and swallow with the saliva into the stomach, certain solid particles which have become detached from the invalid, and which speedily find their way into the blood and poison it. Among these are the following half dozen:

| | | |
|---|---|---|
| Typhus Fever, | Scarlet Fever, | Diphtheria, |
| Typhoid Fever, | Measles, | Small Pox. |

Persons who breathe through the nose only, and avoid swallowing, in the sick chamber, may come out of it unharmed; for the solid particles are arrested in their long, circuitous passage through the dampened channel which leads from the nostrils to the windpipe. An additional safeguard is to sit so that the draught of air may be from you towards the patient; hence, not between him and the fire-place, towards which there is always a current passing, whether there is any fire there or not.

